# Role of [Ca^2+^]_i_ and F-actin on mesothelial barrier function

**DOI:** 10.3389/fphys.2014.00232

**Published:** 2014-06-30

**Authors:** Masayoshi Kuwahara

**Affiliations:** Laboratory of Veterinary Pathophysiology and Animal Health, Department of Veterinary Medical Sciences, Graduate School of Agricultural and Life Sciences, The University of TokyoTokyo, Japan

**Keywords:** actin cytoskeleton, bradykinin, cytosolic calcium, cytochalasin B, F-actin, histamine, pleura, thrombin

## Abstract

The mesothelial layer acts as a biological barrier between the organ and the enveloping serous cavity and may have functions of transport, equilibrium maintenance, and protection. However, the role of the mesothelial cells in regulation of pleural permeability remains essentially undefined. The present study was designed to clarify the effects of bradykinin, histamine, and thrombin on permeability in pleural mesothelial cells. Rat pleural mesothelial cells were cultured *in vitro*, and the permeability of mesothelial monolayers was evaluated by transmesothelial albumin diffusion and electrical resistance measurements. Furthermore, the temporal relationship between changes in the levels of [Ca^2+^]_i_ and the mesothelial permeability was examined. Bradykinin (10 μM), histamine (1 mM), and thrombin (10 U) caused albumin diffusion within 5 min. The electrical resistance of mesothelial monolayer began falling within 5 min of adding each agent. Time and concentration dependency of changes in electrical resistance were almost the same as that in albumin diffusion. Each agent also induced a biphasic elevation of [Ca^2+^]_i_ in pleural mesothelial cells. The concentration-dependency of the [Ca^2+^]_i_ responses were almost similar to that noted for each agent induced albumin diffusion and electrical resistance fall. The increase in permeability occurred with reorganization of F-actin cytoskeleton and increased actin polymerization. These results suggest that the Ca^2+^- dependency of increases induced by these agents in mesothelial permeability have been related to the regulatory role of Ca^2+^ in the F-actin cytoskeletal reorganization in pleural mesothelial cells.

## Introduction

The visceral and parietal pleura are lined by a unicellular layer of mesothelial cells that overlie a discontinuous basal lamina. This mesothelial layer acts as a biological barrier between the organ and the enveloping serous cavity and may have functions of transport, equilibrium maintenance, and protection (Wang, 1985). Changes in pleural permeability, the influx of phagocytic cells, and the leak of serum proteins into the pleural space lead to the development of an inflammatory exudate (Robbins and Kumar, 1987). However, the role of the mesothelial cells in regulation of pleural permeability remains essentially undefined.

We have shown that pleural and pericardial mesothelial cells can respond to histamine (Ito et al., 1995). Histamine elevates intracellular calcium concentration ([Ca^2+^]_i_) and induces F-actin cytoskeletal reorganization in mesothelial cells. Because F-actin-staining studies have shown the regulatory role of [Ca^2+^]_i_ in cytoskeleton assembly in mesothelial cells, we have suggested that changes in [Ca^2+^]_i_ may have relevance to the regulation of mesothelial permeability. Therefore, the present study was designed to clarify the effects of bradykinin, histamine, and thrombin on permeability in pleural mesothelial cells. For this purpose, rat pleural mesothelial cells were cultured *in vitro*, and the permeability of mesothelial monolayers was evaluated by transmesothelial albumin diffusion and electrical resistance measurements. Furthermore, the temporal relationship between changes in the levels of [Ca^2+^]_i_ and the mesothelial permeability was examined.

## Materials and methods

### Reagents, media, and buffers

For the Ca^2+^ measurements we used a N-2-hydroxy-ethylpiperazine-N′-2-ethanesulfonic acid (HEPES) buffer containing (in mM) 10 HEPES, 136.9 NaCl, 5.4 KCl, 1.0 MgCl_2_, 1.5 CaCl_2_, 0.001 EDTA, and 5.5 glucose (HEPES-buffered solution). Dulbecco's modified eagles medium (DMEM), Hanks' balanced salt solution (HBSS), fetal bovine serum (FBS), and trypsin-EDTA were purchased from GIBCO, Grand Island, NY. Bradykinin, thrombin, ionomycin, albumin, cytochalasin B and 1,2-bis(2-aminophenoxy) ethane-N,N,N′,N′-tetraacetic acid (BAPTA) were purchased from Sigma Chemical Co., St. Louis, MO. Fura 2-acetoxymethyl ester (fura 2-AM) was obtained from Dojindo Laboratories, Kumamoto, Japan. Histamine was purchased from Wako, Tokyo, Japan. Other materials and chemicals were obtained from commercial sources.

### Rat pleural mesothelial cell cultures

Rat pleural mesothelial cells were established and maintained, as described previously (Ito et al., 1995). Briefly, adult Wistar Kyoto rats were anesthetized with sodium pentobarbital (50 mg/kg ip) and were immediately killed by exsanguination from a severed abdominal aorta. The complete thoracic wall was removed under sterile conditions and immersed in petri dish for 20 min in HBSS. The parietal pleural surface was scraped repeatedly with cell scrapers. The cells were then seeded into 55-cm^2^ tissue-culture dishes (Corning, Wexford, PA) in DMEM with 10% FBS, 10^5^ U/l penicillin, and 100 mg/l streptomycin. Subsequently, the mesothelial cell cultures were maintained for up to 15 passages at 37°C in a humidified environment containing 5% CO_2_. The cultured cells exhibited the characteristic features of mesothelial cells: a polyhedral, cobblestone morphologic pattern and positive immunohistochemical staining for cytokeratin and vimentin (Kuwahara et al., 1991).

### Transmesothelial albumin permeability

The transmesothelial albumin flux across cultured monolayers of mesothelial cells was measured using an *in vitro* system as described by Rotrosen and Gallin (1986). Monolayers grown on membranes in Millicell-HA inserts (Millipore, Bedford, MA) were gently washed with HBSS and replaced in 24-well culture plates (Costar Cambridge, MA). The monolayers were covered immediately with DMEM containing a trypan blue-albumin complex [36 mg trypan blue and 800 mg bovine serum albumin were dissolved in 100 ml DMEM to yield a stable complex (trypan blue > 99% protein bound as determined by TCA precipitation) with absorption maximum at 590 nm]. To avoid hydrostatic pressure across the monolayer, the fluid levels inside and outside the culture well were equalized. The monolayers were then incubated during the defined time with stimulant at 37°C, 5% CO_2_. At the end of the incubation the monolayer-inserts were carefully removed, and albumin diffusion across the monolayer was quantified by measuring absorbance at 590 nm of the bottom well fluid. Stimulated albumin diffusion was compared with simultaneous controls and expressed as:

% change vs. control = 100 × [Absorbance (test)-Absorbance (control)]/Absorbance (control).

To show the diffusion rate in control (without stimulant), % change albumin diffusion in control was calculated by dividing the absorbance at defined time point by starting (0 time) absorbance.

### Transmesothelial electrical resistance measurements

Transmesothelial electrical resistance was determined using a Millicell-ERS (Millipore, Bedford, MA). Pleural mesothelial cell monolayers grown on membranes in Millicell-HA inserts (Millipore, Bedford, MA) were gently washed with HBSS and replaced in 24-well culture plates (Costar Cambridge, MA). The monolayers were incubated with DMEM at 37°C, 5% CO_2_ for at least 30 min before the start of each experiment. The initial transmesothelial electrical resistance was then determined, and the monolayers were incubated during the defined time with stimulant at 37°C, 5% CO_2_. The resistance of each sample and blank (without monolayers) was measured using Ag/AgCl electrodes placed into both inside and outside Millicell insert. Transmesothelial electrical resistance (TER) was calculated as:

TER (Ohms × cm^2^) = [Resistance (test)-Resistance (blank)]/Effective membrane area (in this study 0.6 cm^2^).

### Measurement of [ca^2+^]_i_

Changes in [Ca^2+^]_i_ were determined as previously reported (Ito et al., 1995). Pleural mesothelial cells were incubated on 25-mm glass coverslips (Matsunami, Tokyo, Japan) in DMEM with 10% FBS. After reaching confluence, the cells were cultured further in serum-free culture medium for 12 h, and then the mesothelial cell monolayers were loaded with fura 2 by incubating them with 2 μM fura 2-AM for 30 min at 37°C in HEPES-buffered solution. Loaded cells were washed in HEPES-buffered solution and maintained in this solution for 20 min at room temperature to allow for complete hydrolysis of fura 2 to the acid form.

The glass coverslip was placed horizontally in a temperature-controlled (37°C) bath that was mounted on Intracellular Ion Analyzer (CAF-110, Japan Spectroscopic, Tokyo, Japan). Fluorescence excitation was obtained from a xenon high-pressure lamp (150 W). Ultraviolet light of alternating 340 and 380 nm (10 nm bandwidth) was obtained with a monochromator equipped with a chopping wheel (400 Hz) placed in front of the monochromator. Fura 2 fluorescence from the cells was imaged with a Nikon UV-Fluor objective lens (× 10). The dichroic mirror was used as a beam splitter to transmit emitted fluorescence (500 nm) into the photomultiplier. The fluorescence signals (340 and 380 nm) and their ratio (340:380 nm) were continuously recorded on a chart recorder. At the end of experimental run, background autofluorescence (the inherent fluorescence emitted from cells, coverslip, and bath at 340 and 380 nm) was obtained by the method of Hallam et al. (1988).

After autofluorescence was subtracted, the changes in [Ca^2+^]_i_ were determined quantitatively by using the following equation: [Ca^2+^]_i_ = *K*_*d*_[(*R*-*R*_min_)/(*R*_max_-*R*)](*Sf*_2_/*Sb*_2_) where the dissociation constant *K*_*d*_ has a value of 224 nM (Grynkiewicz et al., 1985), *R* is the fluorescence ratio within the cells, *R*_max_ is the maximal fluorescence ratio after addition of 40 μM ionomycin in the presence of 1.5 mM CaCl_2_, *R*_min_ is the minimal ratio determined by the subsequent addition of 5 mM EGTA, and *Sf*_2_/*Sb*_2_ is the ratio of fluorescence values at 380-nm excitation determined at *R*_min_ and *R*_max_, respectively.

### F-actin staining

Pleural mesothelial cells were fixed in 3% phosphate-buffered saline (PBS)-formalin for 10 min, and permeabilized with 1% Triton X-100 in PBS for 10 min at room temperature. After two washes with PBS, cells were stained with fluorescein isothiocyanate (FITC)-labeled phalloidin (5 unit/ml in PBS) to localize F-actin for 20 min in a dark room at room temperature. Cells were washed with PBS twice and maintained in PBS. Dishes were mounted on the stage of Leica TCS/NT confocal laser scanning microscope equipped with an Ar–Kr laser. The excitation and emission wavelengths for FITC-phalloidin were 490 and 525 nm. To standardize the fluorescence intensity measurements among experiments, the time of image capture, the image intensity gain, the image enhancement, and the image black level in both channels were optimally adjusted at the outset and kept constant for all experiments.

### Quantification of F-actin content

Mesothelial cells were stained with nitrobenzoxadiazole (NBD)-phallacidin (Molecular Probes, Junction City, OR) and analyzed with a FACScan (Becton Dickinson Immunocytometry System, Mountain View, CA) (Howard and Oresajo, 1985) with following some modifications. In all cases a two-step stain procedure was used. Mesothelial cells suspensions (1 × 10^6^ cells/ml) were incubated at the desired time in HBSS with and without agents; fixed with formalin (3.7% vol/vol) for 15 min at 25°C; and then exposed to a final concentration of 100 μg/ml lysophosphatidyl choline and 1.65 × 10^−7^ M NBD-phallacidin. Stained cells were analyzed by FACScan within 1 h of staining. In all instances the fluorescence histogram of cells yielded a normal distribution, and the fluorescence was recorded as the peak fluorescence channel number. The relative F-actin content is expressed as the ratio of the agents treated peak channel number to the control peak channel number.

### Ca^2+^ chelation and stabilization of the actin cytoskeleton

To show the role of Ca^2+^ and F-actin on the mesothelial permeability, effects of Ca^2+^ chelation by BAPTA (5 mM) and stabilization of the actin cytoskeleton by cytochalasin B (10 μg/ml) were determined for some experiments. The dose of cytochalasin B was selected according to previous study (Kuwahara et al., 1994).

### Statistical analysis

Results were expressed as means ± *SD*. Statistical comparisons were made with the use of the Student's *t*-test or analysis of variance. A value of *P* < 0.05 was considered significant.

## Results

### Changes in albumin diffusion and electrical resistance

**Figure 1:**
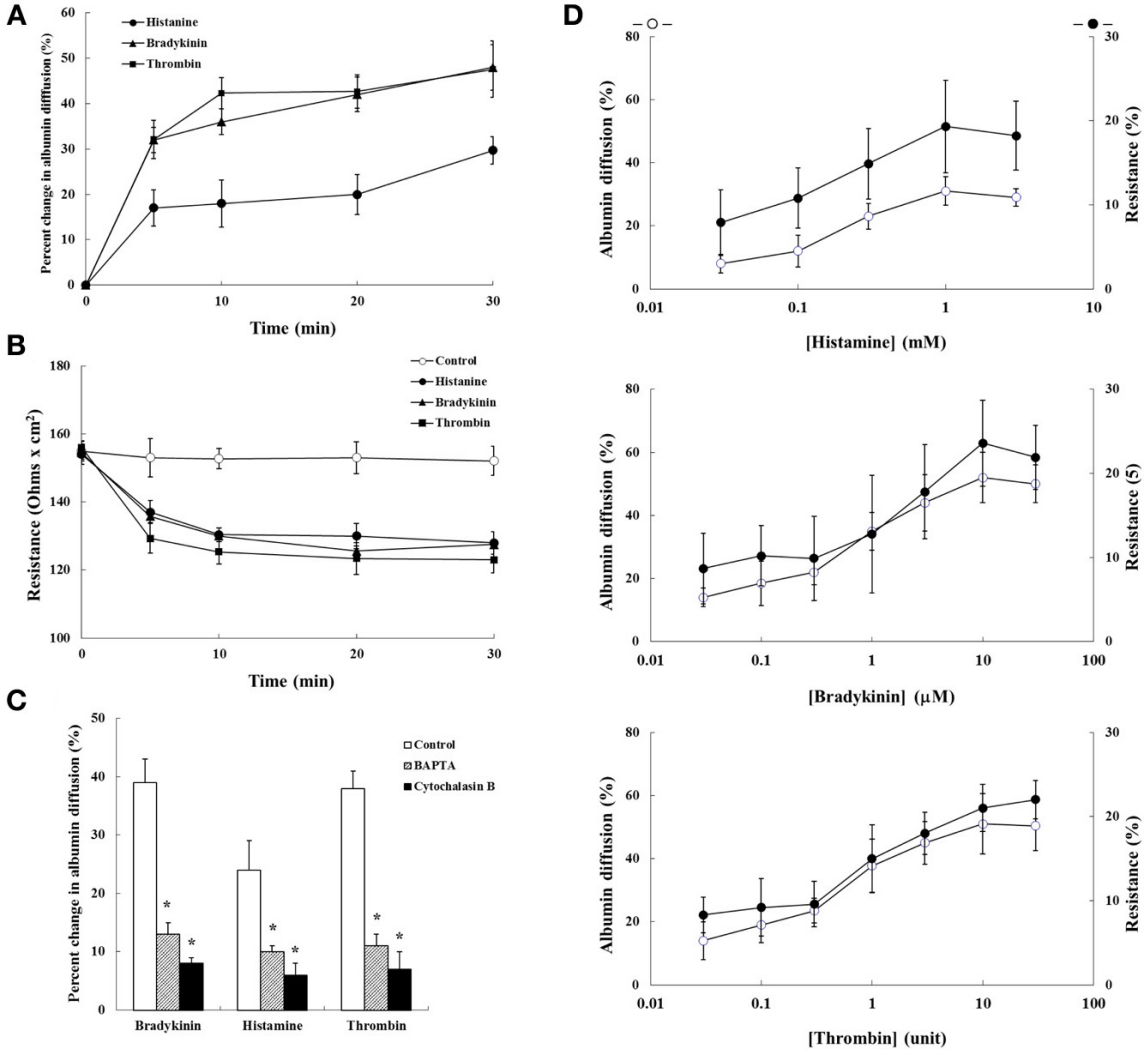
**The effects of bradykinin (10 μM), histamine (1 mM), and thrombin (10 U) on the permeability of mesothelial cell monolayers**. **(A)** Time course of trypan blue-albumin diffusion across the mesothelial cell monolayers, **(B)** time course of transmesothelial electrical resistance of the mesothelial cell monolayers, and **(C)** effects of Ca^2+^ chelation by BAPTA (5 mM) and stabilization of the actin cytoskeleton by cytochalasin B (10 μg/ml). **(D)** Concentration dependency between trypan blue-albumin diffusion and transmesothelial electrical resistance. Values are means ± *SD* of 5 separate experiments. ^*^P < 0.05, significant difference from each control.

The effects of histamine (1 mM), bradykinin (10 μM), and thrombin (10 U) on time course of trypan blue-albumin diffusion across the mesothelial cell monolayers are shown in Figure 1A. All of these agents caused albumin diffusion within 5 min. Albumin diffusion was gradually increased after 5 min exposure. The magnitude of increase in bradykinin and thrombin to albumin diffusion was almost the same, but larger than that in histamine. Albumin diffusion was not induced in control group. The electrical resistance of mesothelial monolayer began falling within 5 min of adding each agent and leveled off after 30 min as shown in Figure 1B. Time dependency of changes in electrical resistance was almost similar to that in albumin diffusion. The effects of Ca^2+^ chelation by BAPTA and stabilization of the actin cytoskeleton by cytochalasin B on albumin diffusion across the mesothelial cell monolayers are shown in Figure 1C. The increase in agents induced albumin diffusion after 10 min incubation was significantly reduced by BAPTA and cytochalasin B treatments. Concentration dependency of changes in both albumin diffusion and electrical resistance of mesothelial monolayer after 30 min incubation is shown in Figure 1D. Each agent induced albumin diffusion and electrical resistance fall in a concentration-dependent manner.

### Changes in [ca^2+^]_i_ in mesothelial cells

**Figure 2:**
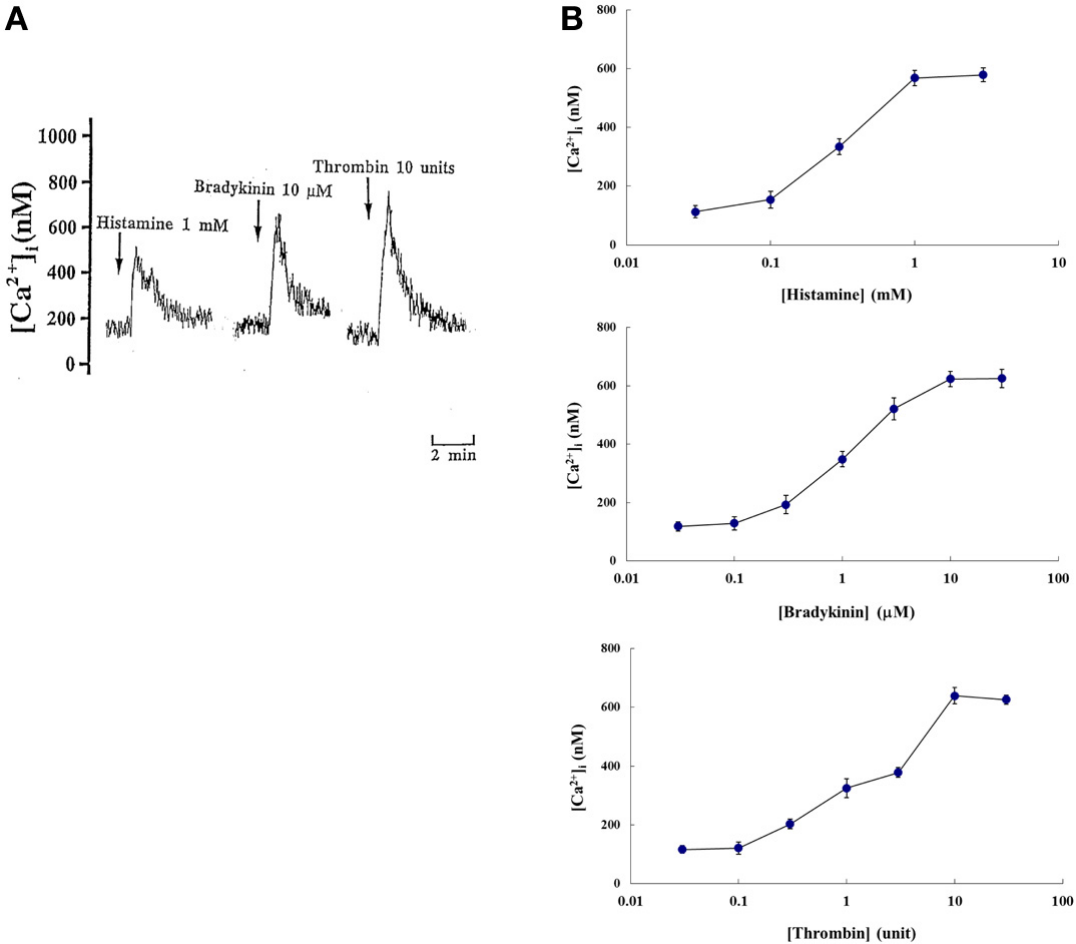
**(A)** Tracings showing effects of histamine (1 mM), bradykinin (10 μM), and thrombin (10 U) on cytoplasmic Ca^2+^ concentration level ([Ca^2+^]_i_) of rat pleural mesothelial cells in the presence of 1.5 mM extracellular Ca^2+^. Addition of each agent induced a biphasic elevation of [Ca^2+^]_i_: a large initial transient component followed by a sustained component in fura 2 loaded pleural mesothelial cells. Each tracing is a representative of 5 independent experiments. **(B)** Dose-response curves showing magnitude of initial transient increase in [Ca^2+^]_i_ in rat pleural mesothelial cells. Each point represent mean ± *SD* of 5~7 separate experiments.

As shown in Figure 2A, histamine (1 mM), bradykinin (10 μM), and thrombin (10 U) induced a biphasic elevation of [Ca^2+^]_i_ in pleural mesothelial cells that consisted of an initial transient component and a following sustained component in the presence of 1.5 mM extracellular Ca^2+^. The peak of initial transient component was appeared within 30 s after stimulation and sustained component was followed. The characteristics of the response were similar for each agent. Each agent elicited the elevation of [Ca^2+^]_i_ in a dose-dependent manner. Concentration-response curves were obtained from the peak values of each initial component of the [Ca^2+^]_i_ response as shown in Figure 2B. These responses of [Ca^2+^]_i_ in the concentration-dependency were almost similar to that noted for each agent induced albumin diffusion and electrical resistance fall.

### F-actin cytoskeletal organization

**Figure 3:**
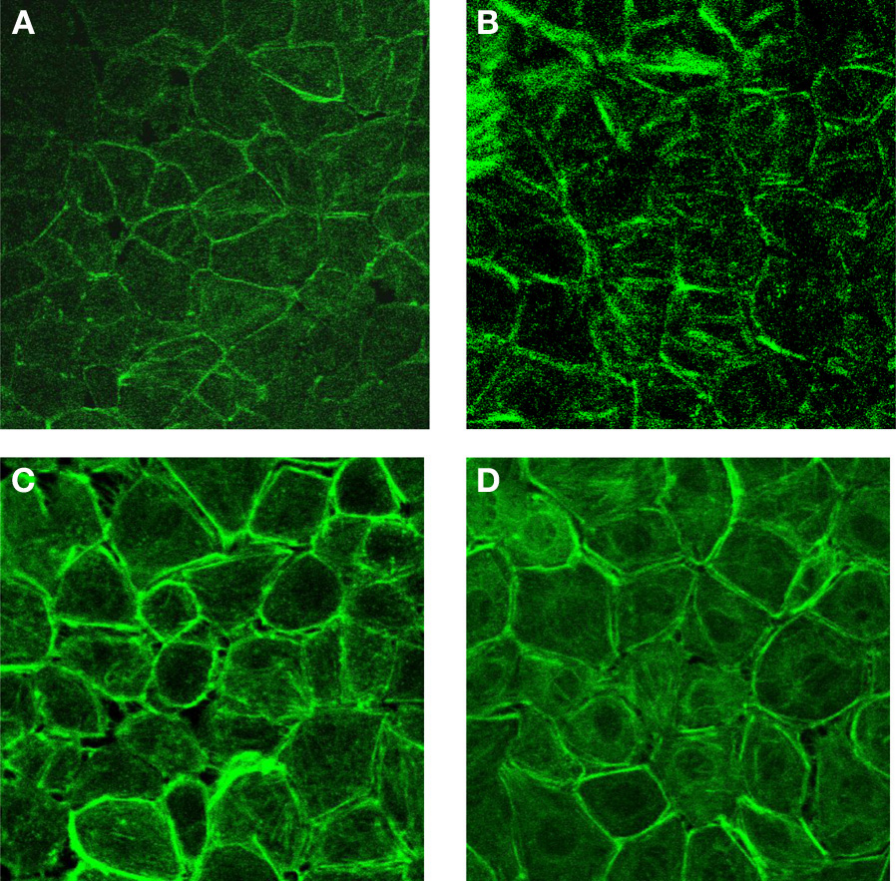
**The effects of histamine (1 mM), bradykinin (10 μM), and thrombin (10 U) on F-actin cytoplasmic distribution in rat pleural mesothelial cells**. Control **(A)**, after 5 min exposure histamine **(B)**, bradykinin **(C)**, and thrombin **(D)**.

The F-actin cytoskeletal organization was studied after 5 min of histamine (1 mM), bradykinin (10 μM), and thrombin (10 U) challenge. The representative F-actin patterns are shown in Figure 3. In the control group, mesothelial cells formed a cobblestone mosaic pattern composed of polyhedral cells (Figure 3A). After 5 min of agent exposure, the cells became elongated compared with the control cells. The density of stress fibers spanning the cells increased in histamine (Figure 3B), bradykinin (Figure 3C), and thrombin (Figure 3D) exposure.

### F-actin content

**Figure 4:**
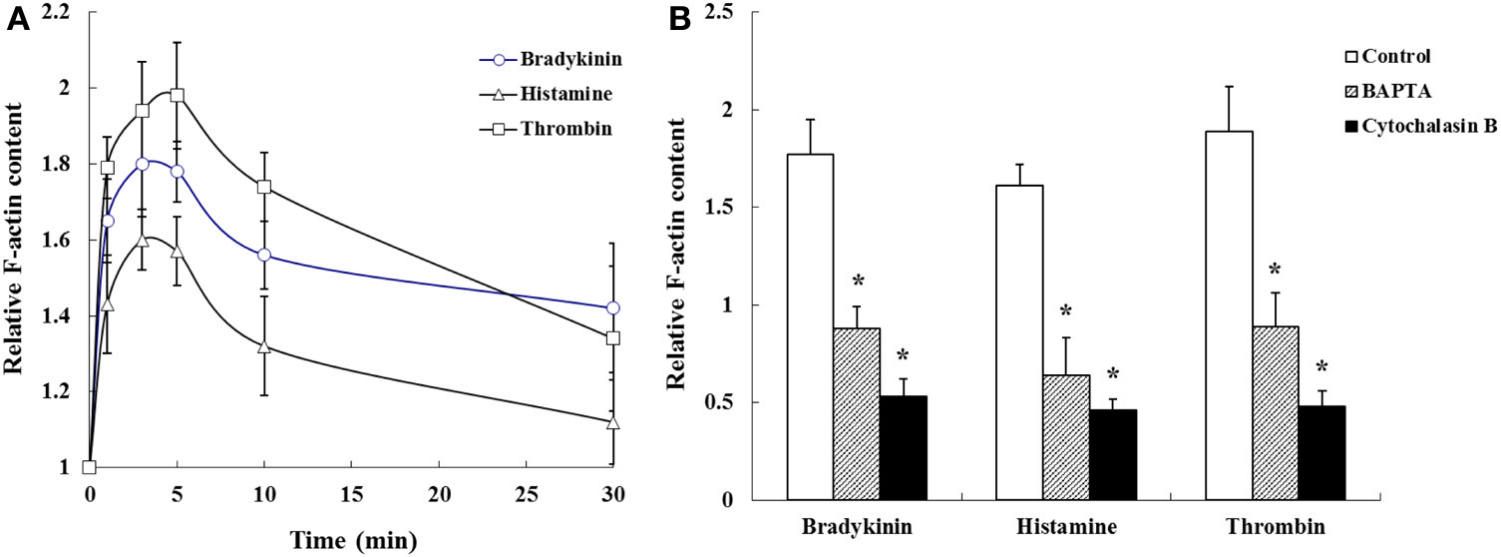
**(A)** Time course of changes and **(B)** effects of Ca^2+^ chelation by BAPTA (5 mM) and stabilization of the actin cytoskeleton by cytochalasin B (10 μg/ml) on histamine (1 mM), bradykinin (10 μM), and thrombin (10 U) induced F-actin content. Values are means ± *SD* of 3 separate experiments. ^*^P < 0.05, significant difference from each control.

Figure 4A shows the time course of change in relative F-actin content during 30 min after agent stimulation. During the first 1 min after stimulation there was a dramatic increase in relative F-actin content that was maximal at 3–5 min and reflects agents induced actin polymerization. After maximal F-actin content was observed there was a decline in relative F-actin content during the next 30 min. These agents induced actin polymerization after 5 min incubation were significantly inhibited by Ca^2+^ chelation or cytochalasin B treatment (Figure 4B).

## Discussion

The present study has demonstrated that bradykinin, histamine, and thrombin increased transmesothelial permeability in cultured pleural mesothelial cell monolayers. The magnitude of increase in mesothelial permeability in response to histamine was smaller than that to bradykinin and thrombin. The Ca^2+^-dependency of the bradykinin-, histamine-, and thrombin-induced increases in mesothelial permeability have been related to the regulatory role of Ca^2+^ in the F-actin cytoskeletal reorganization in pleural mesothelial cells.

We have shown that the mesothelial cells respond to histamine and elevate [Ca^2+^]_i_ via H_1_ receptors pathway (Ito et al., 1995). Hott et al. (1992) have reported that thrombin induces proliferation and chemotaxis of rat pleural mesothelial cells. Bradykinin has also induced [Ca^2+^]_i_ via BK_2_ receptors pathway in human mesothelial cells (Andre et al., 1998), and bradykinin is generated by the serine proteases, kallikreins, at sites of tissue injury and responds in a variety of tissues (Regoli and Barabe, 1980). Furthermore, these agents are known to be potent permeability-increasing agents for endothelial cells (Rotrosen and Gallin, 1986; Alexander et al., 1988; Lum et al., 1989, 1992). Therefore, we have chosen these agents to examine the effects on mesothelial permeability.

Bradykinin, histamine, and thrombin-induced increases in endothelial permeability may be dependent on [Ca^2+^]_i_ elevation and related to the regulatory role of Ca^2+^ in cytoskeleton assembly, structure, and contractility (Berridge, 1987). Rotrosen and Gallin (1986) have reported that concentrations of histamine required to augment monolayer permeability are of the same order of magnitude as those shown to elevate endothelial [Ca^2+^]_i_. Because responses of [Ca^2+^]_i_ in the concentration-dependency were almost similar to that noted for each agent induced mesothelial permeability, our results are in accordance with those observations in endothelial cells. However, because circulating physiological concentrations of these agents may be lower than experimental concentrations in this study, the local paracrine action of these agents in diseased states are more important than their systemic effects in healthy states.

Although the magnitude of the initial transient increase in [Ca^2+^]_i_ induced by higher dosage was almost the same among agents, the magnitude of increase in mesothelial permeability in response to histamine was smaller than that to bradykinin and thrombin. The signal transduction pathways to these agents in mesothelial cells are not fully understood. Three receptor subtypes (BK_1_, BK_2_, and BK_3_) have been classified according to their affinities for bradykinin and to the relative potencies of their agonists and antagonists (Regoli and Barabe, 1980; Farmer et al., 1989; Steranka et al., 1989). Because only the activation of BK_2_ receptors mediates the bradykinin-induced increase in [Ca^2+^]_i_ both endothelial (Morgan-Boyd et al., 1987) and mesothelial (Andre et al., 1998) cells, and phosphoinositide turnover in endothelial cells (Derian and Moskowitz, 1986), it seems that bradykinin-induced elevations of [Ca^2+^]_i_ and permeability in mesothelial cells are dependent on BK_2_ receptors. It is generally thought H_1_ receptor couples G_q/11_ and mediated by protein kinase C. We suggest that angiotensin II-induced actin reorganization in pleural mesothelial cells is dependent on the angiotensin AT_1_ receptor coupled with pertussis toxin-insensitive heterotrimeric G proteins, Rho GTPases and tyrosine phosphorylation pathways (Kuwahara and Kuwahara, 2002). Evidence has suggested G protein activation is a critical step in relaying signals from the receptors to the endothelial contractile machinery (Brock and Capasso, 1989; Voyno-Yasenetskaya et al., 1989; Garcia et al., 1991; Tkachuk and Voyno-Yasenetskaya, 1991). Therefore, one possible explanation of the differences in the magnitude of permeability is the contribution of specific G protein subtypes in activating mesothelial signaling pathways in response to these agents. Another possibility is that selective activation of protein kinase C isoforms or the presence of different degrees of negative feedback pathways may contribute these differences. However, further studies will be necessary to clarify the signal transduction pathways to these agents in mesothelial cells.

The bradykinin, histamine, and thrombin-induced increase of F-actin polymerization occurred at similar time points as that of the permeability increases. The majority of mesothelial cells showed an increased number of centralized actin stress fibers within 5 min of stimulation. Our results show that immediately after stimulation there is a rapid polymerization of actin (F-actin content) followed by a slower depolymerization of actin. Furthermore, agents-induced polymerization is inhibited by in the presence of BAPTA or cytochalasin B. The temporal relationship between cytoskeletal reorganization and permeability increase supports the hypothesis that reorganization of F-actin microfilament is involved in mediating the permeability increase. These findings have important implications for our understanding of the intracellular mechanisms that control the state of actin polymerization and permeability of the mesothelial cells.

Changes in pleural permeability, the influx of phagocytic cells, and the leak of serum proteins into the pleural space lead to the development of an inflammatory exudate. The pleural mesothelial cells produce chemoattractants for fibroblasts, fibronectin (Kuwahara et al., 1991), and for neutrophils, interleukin-8 (Boylan et al., 1992). Fibrinogen and fibrin may serve as chemotaxins (Sueishi et al., 1981), and promote fibroblast adherence, proliferation, and collagen production (Senior et al., 1986). Thrombin may participate in macrophage chemotaxis (Bar-Shavit et al., 1983). Therefore, these factors are also important to maintain the pleural space in healthy and diseased states.

In summary, the effects of bradykinin, histamine and thrombin on permeability in mesothelial cells were studied. The Ca^2+^-dependency of these agents-induced increases in mesothelial permeability have been related to the regulatory role of Ca^2+^ in the F-actin cytoskeletal reorganization in pleural mesothelial cells. This temporal relationship is consistent with the hypothesis that these agents-induced mobilization of Ca^2+^ signals increase in mesothelial permeability.

### Conflict of interest statement

The author declares that the research was conducted in the absence of any commercial or financial relationships that could be construed as a potential conflict of interest.
